# Antique Hunting and Its Philosophy

**Published:** 1918-09-14

**Authors:** 


### THE PRACTITIONER'S HOBBIES AND RELAXATIONS.

### Antique Hunting and its Philosophy.

How strange sounds now this old rubric of our Editor's! Now, when every other practitioner is abroad, and those left at home were working nineteen to the dozen even without influenza epidemics, hobbies must needs, it would seem, be left in the stable to eat their heads off. But not all; some can come out for an airing even now. One, for instance, the amateurship of wild life and of Nature generally, has been pursued at his distant war station and treated of recently in these columns by a colleague who, finding seemingly an additional inspiration of locality, gave us a notable article; a piece of writing worthy of a more permanent place, a little picture, as one (especially in the connection about to be introduced) might say, well worth framing.

Now there is another hobby which in a much more prosaic manner comes to be war-topical. It happens this way. You know that production of ever so many commodities is stopped. Among these commodities is furniture. Try to furnish staff quarters for a new institution and you will very soon hear all about it. What most of the furniture shops are selling now is second-hand goods: let us hope that indeed "the old is better." But what some of us know is that the older is best of all. So much for the immediately practical aspect of my subject; the indirectly practical one is that most people find refreshment in reading about their hobby, even when debarred from practising it, and that many men, especially married doctors, prefer to any other hobby that now known as antiques.

### A Fashionable Foible.

Let us begin, as is proper with enthusiasts, at the philosophy of the subject. The taste for bric-à-brac, then, is surely a poor relation, an attenuated form, of artistic faculty—*vin ordinaire*, low-grade ore, where deep appreciation of the fine arts is the comet vintage and the great mother lode. We do not pretend otherwise. Balzac, who knew everything, made the antique hunter (but, like all that father's children, a mighty one he was) of the "Human Comedy" a professional musician of some little talent. Henry James' unhappy artist born into a Philistine family complains: "I scour our annals without finding the least little sketching grandmother, any sign of a building, or versifying, or *collecting* ancestor." The "suburbs of art" is Mr. George Moore's phrase: the surgeon he tells us of, who, like many another, gets a buhl cabinet or Chinese Chippendale mirror out of the proceeds of an operation, is a great suburban, but has not yet had the freedom of the city; for he can't pick up the right pictures. Perhaps the print connoisseur, again, can't see what Stopford Brooke found to preach about in the "Melencolia." Well, well, granted that it is the lower levels of æstheticism, not the Nauplian heights, whence springs the appetite for bric-à-brac. But there are other sources, for instance the feeling for romance: the miniature of a handsome young officer in Georgian uniform, with its little old ribbon and buckle for a round wrist now dust and ashes, is simply, but very, romantic. Antiques are eminently something to collect, too; and to assemble and house trimly good specimens of anything, be it only empty common match-boxes, is of course an instinct many people own and indulge. Staffordshire cottages are not much more beautiful than chip and paper receptacles for tandstickors, but to get a lot of them together luckily pleases quite a large number of respectable persons—so many fewer purses, we others reflect, to send up the price of lovely *famille rose*! Again, the use of old furniture is even yet a distinction. The sacrifice not-long-married couples of taste make in getting rid of the furnishing company's suite at a loss, and getting in "period" chairs and tables about which they can talk technically, constitutes a *panache*; and that gratifies all humanity, whether the peer with his Garter, or the bearer at a working-class funeral pleasantly conscious of the shoulder cushion tied over his Sunday coat. Then there is the gambling instinct—everyone thinks he will come in for the windfalls and "spot" the forgeries—and the fact that antiques look like old family possessions.

### Why Antique Shops Abound.

All this, with the arrest of production already noted added, is why, at a time when the only ostensible use of articles of luxury is to send them to a Red Cross Sale, antique shops may be seen in dozens. Why, thirty years ago, they could only be seen here and there is a puzzle. The plausible reason is lack of æsthetic enthusiasm in Mid-Victorian times. Thirty years ago the footstool on which once Charles knelt to be beheaded was sold publicly in the provinces for less than ten pounds, while beautiful old Court dresses of like indubitable authenticity went begging. To-day it is hard to say what figure would have power to keep such things out of America. Perhaps here is another reason for the only recent development of the desire for antiquity and rarity in household surroundings: this luxuriousness may be a product of the increase in wealth modern science has created. But if sometimes an indulgence, bric-à-brac is never a gross indulgence. Old things are likely to be finer and more beautiful than new ones for the same reason that in the battle of the books the ancients won all along the line; since, obviously, to have survived a long time posits worth. And thus antique hunting is raising the standard of contemporary production, the modern designer and producer having to meet its competition. No longer, for instance, thanks to one or two firms, is the compound adjective "Tottenham-Court-Road" as applied to furniture *necessarily* a term of

**Practitioner's Hobbies and Relaxations**—(*continued*).

reproach. Better, of course, than the loveliest antique would be the contemporary classic as yet uncrowned; that rarely appears and is always unrecognisable at first. But some bids for the custom of the well-to-do housetaker are more or less damnable. Ingenuity in copying old models does not atone for the monkeyism of that practice. Further, if everyone with an income of five hundred a year and over wants antiques, then demonstrably antiques must be made as well as found; parts of some must be assembled with parts of others, like, I fear, burials from a dissecting-room; old parts must be joined with new middles, like torpedoed ships; worst of all, wholesale faking becomes a clandestine industry.

COLLECTING MODERATELY AN ELEVATING HOBBY.

Collecting in moderation must always, however, remain an elevating hobby, better for the soul than playing cards, and not much worse for the body (Balzac said you needed the "legs of a stag") than playing golf. Philistines never collect anything, except stamps. The very dealer himself enjoys his business. It is one to be prescribed to couples in the latter months of engagement, for contemporary engaged couples go about their furnishing as the Victorian young man went about singing before company—namely, by the light of Nature. If they listen they may save themselves money, and later regret at having to translate to bedrooms unsuitable furniture which happens to be unsaleable; for the present position, when anything except very heavy pieces will sell, will not, fortunately, last for ever. They may also increase their happiness, for bric-à-brac is one of the few hobbies entirely shareable with one's wife. Wives are no kindly critics of masculine versatility, but there are some who will learn to french polish, which is more than joiners must do. Bric-à-brac has distinct feminine affinities. The superficial æstheticism it gratifies is almost universal amongst women, because of their primal instinct to attract. Bric-à-brac is an affair of the house interior; it concerns the lining of the nest, and so appeals to the wife and mother. So while (for other reasons, to be sure) acting and novel-writing are the arts women do well in, and long-distance swimming is the sport, bric-à-brac is the feminine hobby. There are women among its authorities, which is rare. Henry James had in some ways a feminine mind; now he often brings in bric-à-brac, and in "The Spoils of Poynton" the enthusiast who almost starved to replace wall-paper with tapestry was a woman. Yes, nearly any wife will sympathise with the taste for antiques, perhaps even too whole-heartedly for the cause of economy. But what of the children? With them rests, of course, the future of the little collection. In this late-marrying epoch, they will not be likely to begin house-keeping before their parents' is broken up, so that there will be no great obstacle to the furniture becoming an heirloom. But their taste may only too probably differ from yours. They may be cubists or modernists, or more likely worshippers of the passing fashion, and only with added years come to see that the cabriole curve of a Queen Anne table-leg, having been thought beautiful for over a century, has real claim to its reputation. But the thought of that contingency must be put up with: there is no more irritating sophistry than making out one's plan all profit and no loss.

CADUCEUS.

